# Bibliometric and visualised analysis on non-invasive cerebellar stimulation from 1995 to 2021

**DOI:** 10.3389/fnins.2023.1047238

**Published:** 2023-03-31

**Authors:** Lin He, Qi-Fan Guo, Yu Hu, Hui-Xin Tan, Yi Chen, Chen-Han Wang, Tian-Yu Zhou, Qiang Gao

**Affiliations:** ^1^West China Hospital, Sichuan University, Chengdu, China; ^2^Department of Rehabilitation Medicine, West China Hospital, Sichuan University, Chengdu, China; ^3^Department of Rehabilitation Medicine, The Third People's Hospital of Chengdu, Chengdu, China; ^4^Department of Rehabilitation Medicine, The Affiliated Hospital of Qingdao University, Qingdao, China; ^5^MSk Lab, Faculty of Medicine, Imperial College London, London, United Kingdom

**Keywords:** cerebellum, transcranial magnetic stimulation, transcranial direct current stimulation, bibliometrics, non-invasive cerebellar stimulation

## Abstract

**Background:**

The non-invasive cerebellar stimulation (NICS) is a neural modulation technique, which shows the therapeutic and diagnostic potentials for rehabilitating brain functions in neurological or psychiatric diseases. There is a rapid growth in the clinical research related to NICS in recent years. Hence, we applied a bibliometric approach to analyze the current status, the hot spots, and the trends of NICS visually and systematically.

**Methods:**

We searched the NICS publications from the Web of Science (Wos) between 1995 and 2021. Both VOSviewer (1.6.18) and Citespace (Version 6.1.2) software were used to generate the co-occurrence or co-cited network maps about the authors, institutions, countries, journals, and keywords.

**Results:**

A total of 710 articles were identified in accordance with our inclusion criteria. The linear regression analysis shows a statistical increase in the number of publications per year on NICS research over time (*p* < 0.001). The Italy and University College London ranked the first in this field with 182 and 33 publications, respectively. Koch, Giacomo was the most prolific author (36 papers). The journal of Cerebellum, Brain stimulation and Clinical neurophysiology were the most three productive journals to publish NICS-related articles.

**Conclusion:**

Our findings provide the useful information regarding to the global trends and frontiers in NICS field. Hot topic was focused on the interaction between the transcranial direct current stimulation and functional connectivity in the brain. It could guide the future research and clinical application of NICS.

## Introduction

The cerebellum is well known to play an important role in movement execution, motor control, cognitive operations, and social/affective regulation, and it is closely connected with the cortical areas through the cerebellar-thalamic-cortical circuit (Stoodley and Schmahmann, 2009; Koziol et al., 2012). To be specific, it has the close connections between the primary motor cortex, premotor, prefrontal, and other cerebral regions (França et al., 2018). In the field of non-invasive brain stimulation (NIBS), the non-invasive cerebellar stimulation (NICS) is an exciting new area in modulating the excitability of remote cortical regions, the functions of cerebellar-cerebral loops and cerebral-spinal loops (van Dun et al., 2017; Wessel and Hummel, 2018). The NICS technique, as a relatively new type of rehabilitation treatment, is a non-invasive and non-painful method for modulating cerebellar or brain excitability (Wessel and Hummel, 2018; Manto et al., 2021a). Numerous studies have confirmed the strong feasibility and good safety of NICS in humans (Naro et al., 2016a). For the NICS, it mainly contains the transcranial direct current stimulation (tDCS; anodal or cathodal), tACS (transcranial alternating current stimulation) and transcranial magnetic stimulation (TMS; single or repetitive (rTMS); theta burst stimulation (TBS)) (Tomlinson et al., 2013; Manto et al., 2021b). These NICS techniques are growingly gaining the interests in both research and clinical application, particularly in the field of neurorehabilitation (Wessel and Hummel, 2018). In recent years, clinical research in this field is growing, and hundreds of publications investigates the clinical utilisation of NICS in neurologic diseases. For instance, several studies stated that both cerebellar TMS and tDCS could effectively reduce dysfunctions in cerebellar ataxias, cerebral stroke, and Parkinson's disease (Lo et al., 2009; França et al., 2018; Koch et al., 2019). It is not only with effects on the motor functions, visually guided tracking tasks, motor learning and adaptation, but also for the cognitive and affective functions (Langguth et al., 2008; Ferrucci and Priori, 2014; Celnik, 2015). As the breadth of both experimental and clinically established indications for NICS expands, and the application of NICS techniques (such as cerebellar rTMS/iTBS/cTBS and cerebellar tDCS) have also evolved. Although there exist systematic review and meta-analysis that have provided a general overview of specific research questions in NICS, none have summarised the large quantities of literatures to present the state of intellectual structure and emerging trends in the field of NICS. Moreover, these new applications of NICS have also prompt the number of publications in this field. Hence, such a significant growth in publications requires new approaches to review and analyze trends within knowledge domains.

Bibliometric analysis is a quantitative method for analysing the published literatures as well as visualising the trends of research in a given field (Ellegaard and Wallin, 2015; Thompson and Walker, 2015). It can provide a broad synthesis of a research field and how it has changed over time. Moreover, it not only helps researchers and clinicians to have a clearer overview of the research on a given topic, but also it can predict research hotspots and trends (Donthu et al., 2021). An increasing number of researchers and academic institutions devote themselves to explore this specific research field and to publish noninvasive cerebellar stimulation articles over the last decade. Therefore, it is important for researchers or clinicians to understand the present and future direction in this given field. Nevertheless, no bibliometric study has been conducted to provide the progress and the whole-field trends of this field. Thus, it is necessary and meaningful to take a bibliometric analysis of literatures in NICS. In this study, we applied the Citespace and Vosviewer to systematically analyze the NICS publications over the past three decades based on the Web of Science (WoS). Our primary purpose was to detect the research hotspots and emerging trends of key research themes by using networks of co-occurring keywords. Our secondary purpose was to offer clinicians and researchers with a measure of the research network (countries, institutions, authors, and journals).

We hope our study will answer the following research questions (RQ).

RQ1. What is the publication trend for NICS?
RQ2. Which are the most influential authors and primary contributing institutions, countries, and journals for NICS?
RQ3. What are the potential collaborators (author, institutions, countries) for NICS?
RQ4. What are the major themes and frontier topics for NICS?

## Materials and methods

### Source of data and search strategy

We retrieved and downloaded the published papers from the science citation index expanded (SCI-Expanded) of WoS database on 15th June 2022, and the time span was set from inception to the end of 2021. In order to take a comprehensive searching, the search strategy was designed as follow: TS = ((TBS^*^) OR (iTBS^*^) OR (cTBS^*^) OR (TMS^*^) OR (“transcranial magnetic stimulation”) OR (rTMS^*^) OR (“repetitive transcranial magnetic stimulation”) OR (“theta burst^*^”) OR (“paired pulse magnet^*^”) OR (“paired pulse tms”) OR (“paired associative^*^”) OR (“Transcranial Magnetic^*^”) OR (“repetitive transcranial^*^”) OR (“repetitive TMS”) OR (tDCS^*^) OR (“transcranial direct current stimulation”) OR (“non-invasive brain stimulation”) OR (“transcranial ultrasound stimulation”) OR (“Transcranial photobiomodulation”) OR (neurofeedback^*^) OR (TUS^*^) OR (tPBM^*^)) AND TS = “cerebell^*^”.

### Inclusion criteria

**Figure 1:**
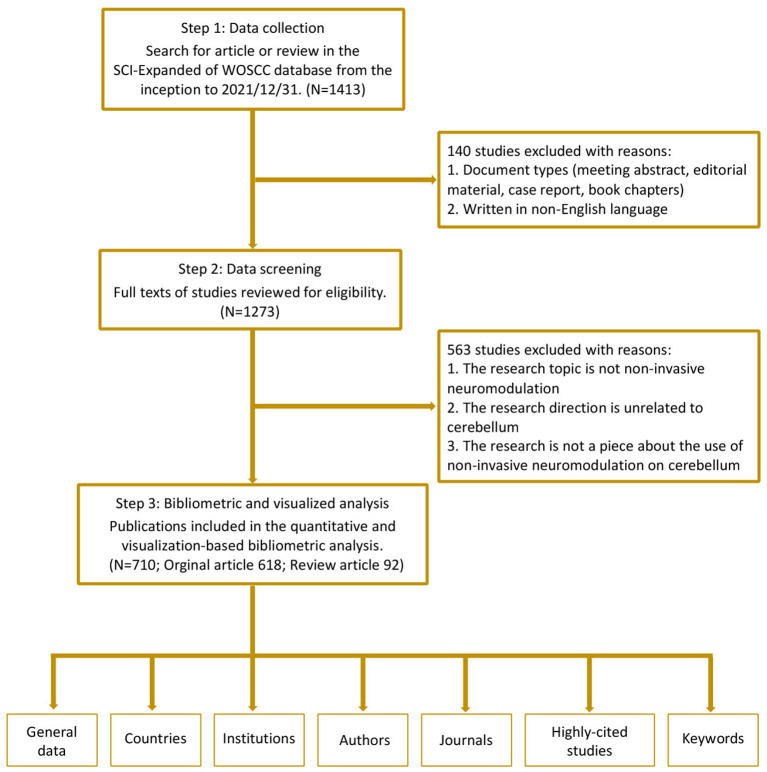
Flow chart of the bibliometric search and NICS inclusion.

All publications should meet the following inclusion criteria: (1) papers were related to the NICS topics; (2) the document types were the original articles and reviews; (3) the publication language was restricted to English. The flow chart of article selection is shown in Figure 1.

### Statistical analysis

After searching from WoS core collection, we obtained the general information, including the number of publications, publication years, H-index, keywords, the total and annual citation for each paper. And then these bibliometric data were imported into EndNote X9 (Bld 7072, Thomson Research Soft, Stamford, CA, USA) and Microsoft Office Excel. In present study, we explored the performance analysis (e.g., primary countries, contributors, institutions, and journals), science mapping analysis (e.g., collaborations, research themes, and trends) and clustering. For the scientific map analysis, it contained citation analysis, co-citation analysis, co-word analysis, and co-authorship analysis (Donthu et al., 2021). Citation analysis mainly revealed the intellectual structure of a research field, and co-word analysis was frequently applied to explore the existing or future relationships among topics in a research field (Podsakoff et al., 2005; Emich et al., 2020). Co-authorship analysis was performed to examine the relationships among authors and their affiliations on the development of the research field (Acedo et al., 2006). VOSviewer (1.6.18) was used to perform the co-authorship network analysis of countries, institutions, authors, and co-cited network maps of journals. The units of measures were country, institution, author, and journal. In the network graphs, the node size in the VOSviewer map represented the number of published articles, and the link between the nodes indicated the relationships or cooperation strength. Citespace (Version 6.1.2) was applied to obtain the co-occurring keywords network (co-word analysis) and burst keywords, predicting the cutting-edge knowledge and research trends. The co-occurrence networks represented how frequently variables appear together, and the colour of each annual ring mean the publication year or clusters, and the cluster labels were generated from noun phrases of the keyword lists of articles cited in each cluster. When examining the burst of keywords, it indicated that a particular keyword is associated with a surge of citations. Moreover, we applied the Microsoft Office Excel to perform the linear regression analysis to evaluate the time trend of annual publications.

## Results

### General data

After the initial searching, 1413 publications were identified. We excluded the irrelevant topic, non-English writing, meeting abstract, editorial material and book chapters, and a total of 710 articles were finally included into analysis. The type of most publications was the original article, accounting for 87% (Figure 1).

### Output and growth trends of publications analysis

**Figure 2:**
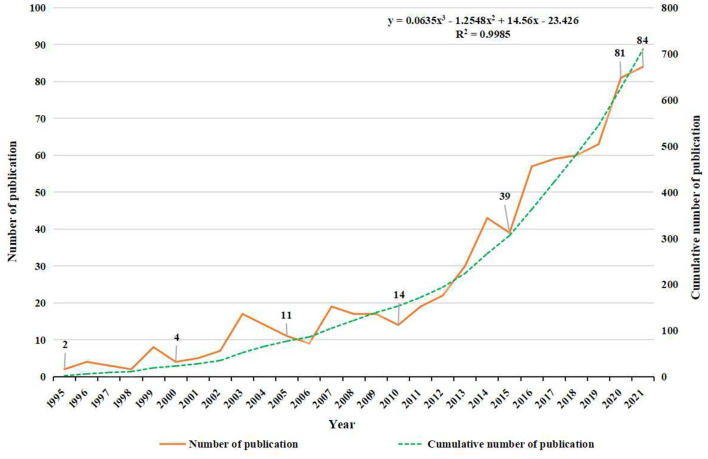
Trend of publication outputs from 1995 to 2021 on NICS.

As shown in Figure 2, the annual publication output had an obviously increasing trend from 2 in 1995 to 84 in 2021. The overall trend is positive, and it is divided into two stages. In the first stage from 1995 to 2011, there was a relatively slow increase, and the average number of publications was less than 20 annually. The second stage (2012-2021) was the period with the highest growth, and the year of 2021 reached a peak of 84 publications. Within this study, the linear regression analysis showed that the publications per year positively correlated with the publication year (*R*^2^ = 0.998, *p* < 0.001) (Figure 2).

### Distribution of countries

**Figure 3:**
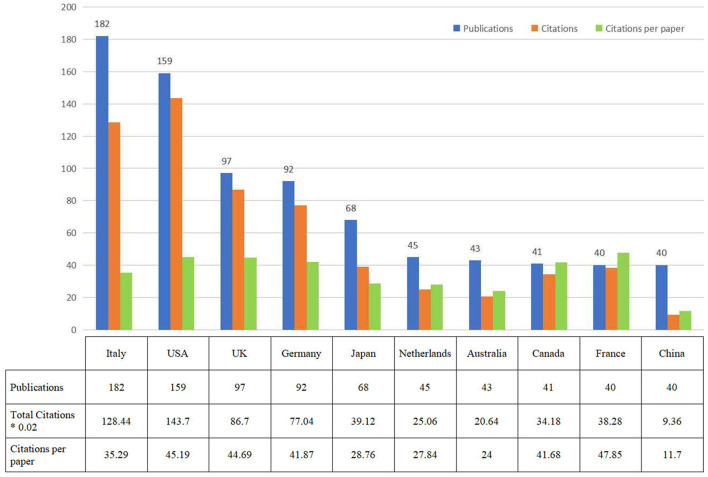
The number of publications, total citations, and citations per paper in top 10 countries.

A total of 45 countries published 711 papers on NICS-related research. In terms of publications, citations, and average citation, the top 10 countries are displayed in Figure 3. During the period of 1995 to 2021, Italy had the highest number of publications (*n* = 182, 25.6%), followed by the United stated (USA) (*n* = 159, 22.4%), the United kingdom (UK) (*n* = 97, 13.6%), Germany (*n* = 92, 12.8%) and Japan (*n* = 68, 9.6%). These countries were the dominant contributions in this field, accounting for more than 80% in all NICS publications. The top three ranked countries by total citation were the USA (7,185), Italy (6,422), and UK (4,335).

**Figure 4:**
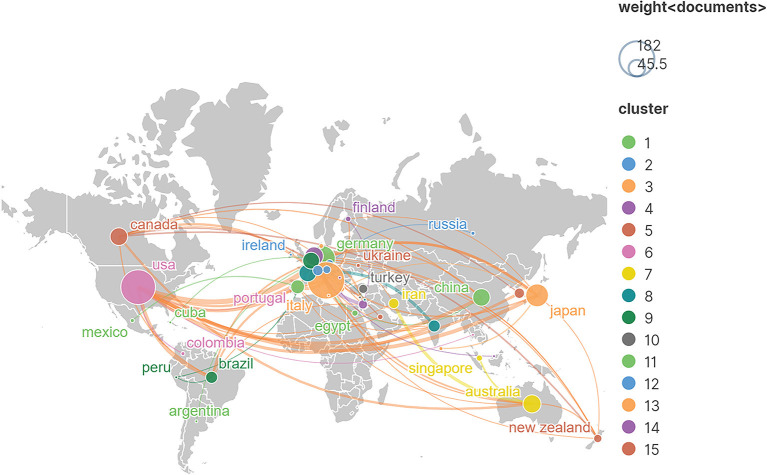
The co-authorship network visualisation map of countries in non-invasive cerebellar stimulation research. In the network map, a node represents a country, and node size indicates the number of publications. A line between two nodes associates with the cooperation relationship.

In the collaboration network of countries, the size of nodes represents the number of published papers, and the thickness of link between two nodes means the closeness of cooperation between two countries or regions. Figure 4 shows that the USA developed the extensive and close research partnerships with other countries, followed by Italy, UK, Germany, Belgium, and Netherlands. Moreover, the important research cooperators of the USA were Italy and UK.

### Distribution of institutions

**Figure 5:**
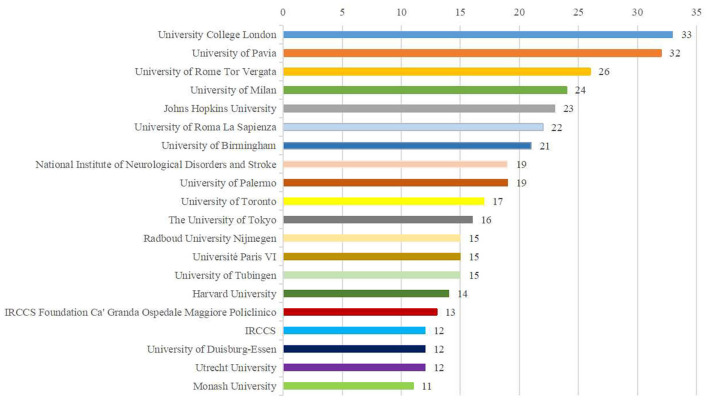
The top 20 most productive institutions in the NICS field.

**Figure 6:**
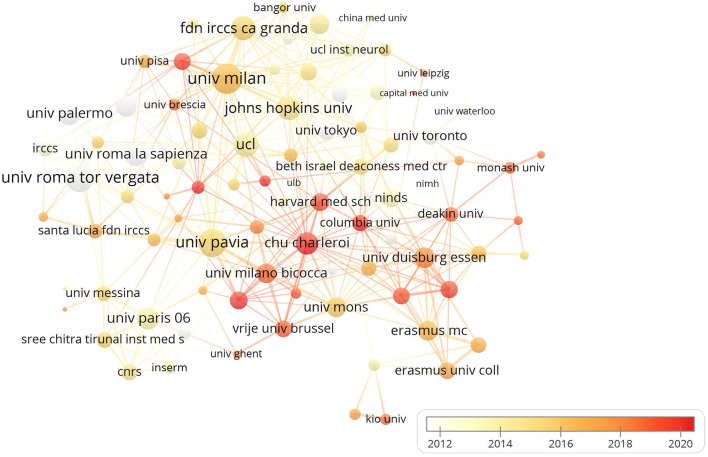
The co-authorship network visualisation map of institution for NICS research. Institutions were coloured according to the appearance for the average time. The yellow colour represented early stage and red colour represented late stage.

The 10 institutions with the highest number of publications are presented in Figure 5. University College London were the most prolific institutions (*n* = 33), followed by the University of Pavia (*n* = 32) and University of Rome Tor Vergata (*n* = 26). Amongst the top 10 institutions, 5 research institutions were located in Italy, 2 in UK and the USA, and 1 in Canada, respectively. In terms of total citations, the top three were the Johns Hopkins University (2,058), University College London (1,482) and the University of Rome Tor Vergata (1420), showing in Table 1. Regarding to the average citation, Johns Hopkins University (89.48) and the University of Toronto (73.41) were the leading institutions, followed by the National Institute of Neurological Disorders and Stroke (68.26) (Table 1). In the co-authorship network analysis among 893 institutions, there were close and extensive connecting lines between top 10 institutions, especially in the University of Milan, University of Rome Tor Vergata, the University of Pavia, Johns Hopkins University and University College London, as shown in Figure 6.

**Table 1:** The number of publications, total citations, and average citation in top 10 institutions.

**Rank**	**Institution**	**Publications**	**Citations**	**Average citation/ publication**
1	University College London	33	1482	44.91
2	University of Pavia	32	859	26.84
3	University of Rome Tor Vergata	26	1420	54.62
4	University of Milan	24	1114	46.42
5	Johns Hopkins University	23	2058	89.48
6	University of Roma La Sapienza	22	753	34.23
7	University of Birmingham	21	1171	55.76
8	National Institute of Neurological Disorders and Stroke	19	1297	68.26
9	University of Palermo	19	1026	54.00
10	University of Toronto	17	1248	73.41

### Distribution of journals

**Figure 7:**
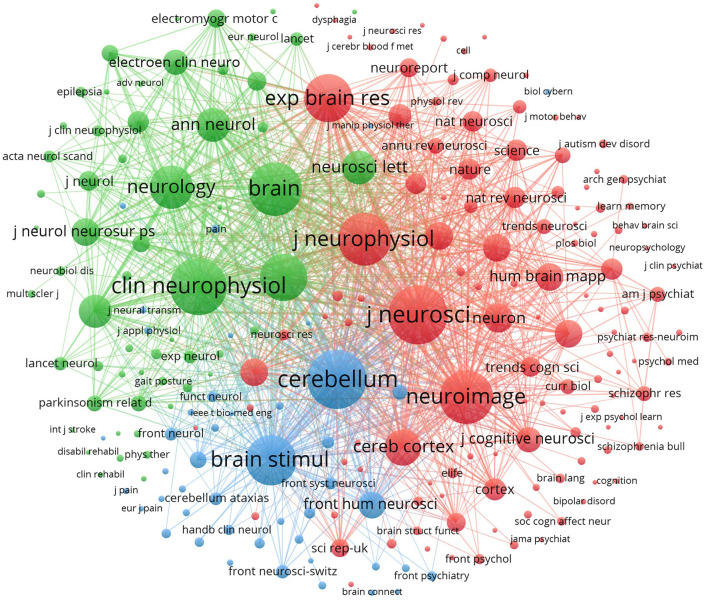
Co-citation network map of journals in NICS field between 1995 and 2021.

From 1995 to 2021, the number of 181 journals published papers related to non-invasive cerebellum modulation, and there were 11 journals with the number of publications more than 15. Table 2 lists the top 10 journals with the most productive and impactful. The Cerebellum was the most popular journal for publishing NICS-related articles (*n* = 97, IF = 3.847), followed by the Brain stimulation (*n* = 37, IF = 8.955), Clinical neurophysiology (*n* = 32, IF = 3.708), Frontiers in human neuroscience (*n* = 23, IF = 3.169) and Plos one (*n* = 20, IF = 3.240). These top-5 journals contributed to papers about 29.4% of the total number of publications. Moreover, the journal of Cerebellum was the greatest number of total citation (*n* = 2327), and the Journal of neuroscience had the highest average citation per paper (Table 2). As shown in Figure 7, the top three co-cited journals were identified by the Vosviewer, including the journal of neuroscience (1,852), Cerebellum (1,652) and the journal of neurophysiology (1,554), respectively.

**Table 2:** The top 11 journals that published articles on NICS research.

**Rank**	**Journal**	**Publications**	**IF**	**JCR**	**OA**	**Citations**	**Average citation/ publication**
1	Cerebellum	97	3.847	Q2	No	2327	23.99
2	Brain stimulation	37	8.955	Q1	No	1166	31.51
3	Clinical neurophysiology	32	3.708	Q2	No	1341	41.91
4	Frontiers in human neuroscience	23	3.169	Q2/Q3	Yes	376	16.35
5	Plos one	20	3.240	Q2	Yes	366	18.30
6	Neuroimage	19	6.556	Q1	Yes	1119	58.89
7	Experimental brain research	18	1.972	Q4	No	529	29.39
8	Journal of neurophysiology	16	2.714	Q2/Q3	No	891	55.69
9	Journal of neuroscience	16	6.167	Q1	No	1074	67.13
10	European journal of neuroscience	15	3.386	Q3	No	618	41.20
11	Scientific reports	15	4.379	Q1	Yes	171	11.40

### Distribution of authors

During the past three decades (1995–2021), a total of 2,576 authors published the relevant papers on the field of NICS. Amongst the top 10 prolific authors presented in Table 3, the average number of published papers was 14 at least, and 60% authors were from Italy. In terms of publication output, the highest-ranking author was Giacomo Koch from the University of Rome Tor Vergata (publications = 36), followed by John C. Rothwell from University of London College (publications = 35) and Alberto Priori from University of Milan (publications = 22). In terms of citation, the top three cited authors were Pablo A. Celnik, John C. Rothwell and Giacomo Koch with 1,884 citations, 1,741 citations and 1,644 citations, respectively (Table 3).

**Table 3:** Top 10 active authors on NICS research.

**Rank**	**Author**	**Country**	**Institution**	**Documents**	**Citations**	**Average citation/ publication**	**H-index**
1	Giacomo Koch	Italy	University of Rome Tor Vergata	36	1644	45.67	22
2	John Rothwell	UK	University of London College	35	1741	49.74	152
3	Alberto Priori	Italy	University of Milan	22	1067	48.50	33
4	Carlo Caltagirone	Italy	University of Rome Tor Vergata	20	1003	50.15	48
5	Pablo Celnik	USA	Johns Hopkins University	20	1884	94.20	38
6	Egidio D'angelo	Italy	University of Pavia	20	663	33.15	19
7	Mario Manto	Belgium	The University of Mons	20	669	33.45	38
8	Roberta Ferrucci	Italy	University of Milan	19	992	52.21	32
9	Chris Miall	UK	University of Birmingham	16	965	60.31	52
10	Massimiliano Oliveri	Italy	University of Palermo	14	772	55.14	44

**Figure 8:**
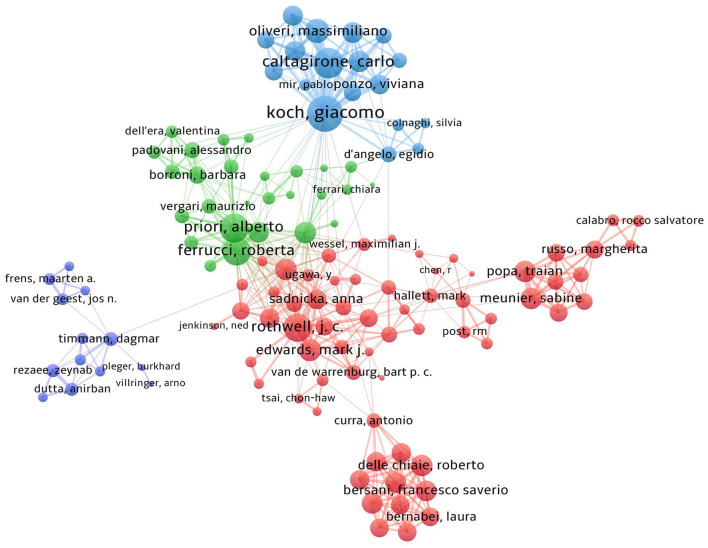
The co-authorship network map of authors in NICS field. Node size represents the number of publications, and the line between any two nodes indicates the cooperation strength.

Figure 8 shows the whole cooperative network maps between each author on the NICS research, in which each node represents an author. A larger node means the more publication outputs, and the lines between different nodes indicate the collaboration between authors. As presented in Figure 8, there were four clusters of authors. The author groups were centred on Giacomo Koch, John Rothwell, Alberto Priori and Roberta Ferrucci, constituting the largest cooperative network.

### Co-occurring keywords and cluster analysis

**Figure 9:**
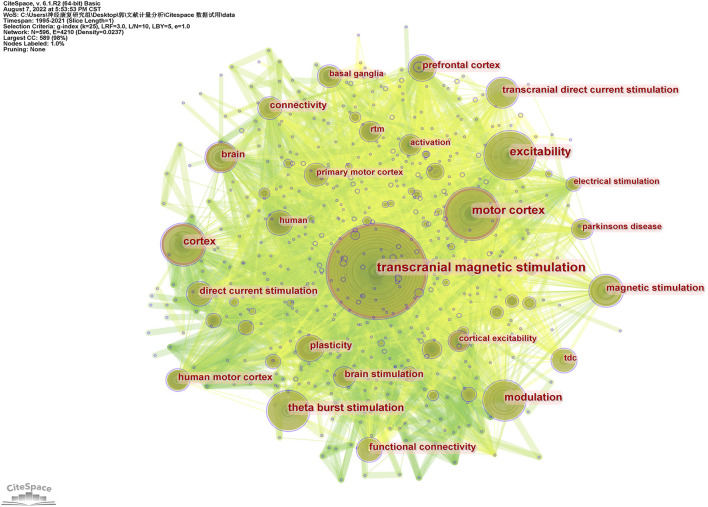
Analysis of keywords related to publications on NICS field. The co-occurrence network of keywords related to NICS field.

The analysis of keyword co-occurrence describes the hot and frontier topics, and the centrality of keywords reveals the role and influence of corresponding research content in a research field. 596 keywords were identified by using CiteSpace, while 30 keywords occurred more than 30 times. From 1995 to 2021, hot keywords in high-frequency and centrality were “transcranial magnetic stimulation” (frequency: 344, centrality: 0.12), “motor cortex” (frequency:161, centrality: 0.13), “theta burst stimulation” (frequency: 101, centrality: 0.04) and “transcranial direct current stimulation” (frequency: 84, centrality: 0.04) (showing in Figure 9).

**Figure 10:**
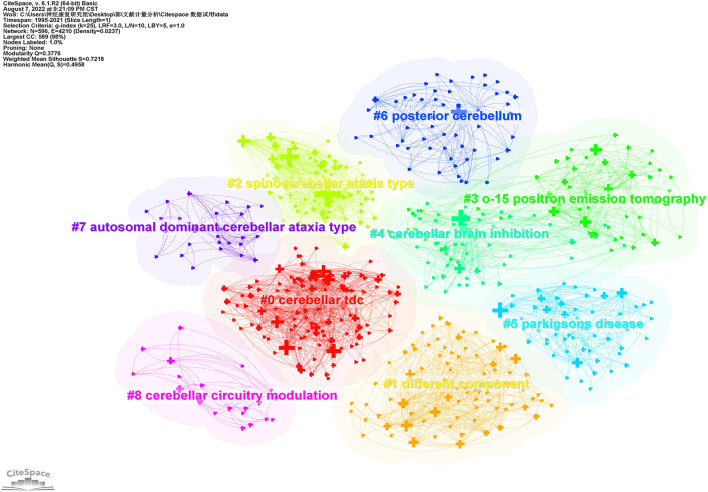
The keyword co-occurrence cluster map to NICS field.

Cluster analysis of co-occurrence keywords can be used to reveal the main topics in this field. A total of 9 clusters with a Q-value of 0.3776, and the silhouette value for each cluster was over 0.5, indicating that clustering results were reasonable and reliable. As presented in Figure 10, the largest cluster #0 was “cerebellar tDCS,” followed by #1 “different component,” #2 “spinal-cerebellar ataxia type” and #3 “position emission tomography.” Clusters #0,#1,#3,#4,#5,#6,#8 mainly described the NICS techniques for the neurological diseases. Clusters #2 and #7 mainly summarised the application of NICS for cerebellar ataxia diseases.

### Keywords with citation bursts

**Figure 11:**
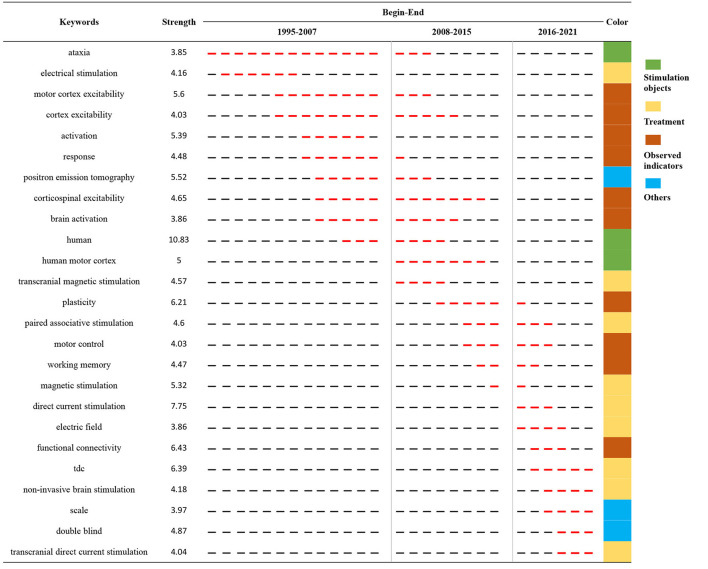
The top 25 keywords with the strongest citation bursts on NICS field between 1995 and 2021. The red segment of the black line denoted the burst duration of a keyword.

Burst keywords were the indicators of hotspots and emerging trends in a given field. Figure 11 displays the top 25 keywords with the strongest citation bursts. Among them, the ataxia (1995-2010) was the first hot keyword, along with the longest duration. Between 1996 to 2011, keywords with citation bursts were “electrical stimulation,” “motor cortex excitability,” “activation,” ‘response,” “position emission tomography,” “corticospinal excitability,” “brain activation,” “human,” “human motor cortex,” and “transcranial magnetic stimulation.” In the past five years (2016-2021), the latest hot keywords were direct current stimulation, electric field, functional connectivity, tDCS, non-invasive brain stimulation, double blind, and transcranial direct current stimulation.

## Discussion

### Summary of findings

The current study provides an insight into the research status, while identifying the hot spots and emerging trends of non-invasive cerebellar modulation. In the past 26 years, the annual number of published papers has continued to increase within time. 1995–2012 was the initial period with a relatively slow growth, and there is the average lowest number of publications annually (*n* < 20). By the date of our search, the first paper related to NICS was published by Hashimoto and Ohtsuka (1995), who investigated the effects of TMS over the posterior cerebellum on the control of saccadic eye movements. Moreover, most of NIBS techniques were primarily be introduced to modulate the excitability of specific brain areas in several psychiatric disorders, such as left or right dorsolateral prefrontal cortex (Pridmore and Belmaker, 1999; McLean, 2019; Matsuda et al., 2021). Therefore, in the early stage, the cerebellar stimulation techniques have not yet developed into a sufficiently mature technology, potentially leading to the limited applications in clinical or research settings. During the period of 2013 to 2021, the rate of development shows the significant increase with a peak in 2021. These findings reveal that an increasing number of researchers have been devoted themselves to the field of NICS, and this may be because of utilising the cerebellar stimulation in the neurological conditions. These results support that the field of NICS has received the increasing attentions. Based on the above analysis of publication trends, we predicted that the field of NICS is a potential research area, since there may be still some crucial issues that have not been solved. Therefore, it is worthwhile for researchers to conduct more in-depth studies in this field.

Based on the Bradford's law (Venable et al., 2016), the top 10 journals could be considered as the core journals in this area, showing in the Table 2. Notably, the impact factor (IF) of top 10 journals were distributed between 1.00 and 9.00, and the journal of Brain Stimulation ranked the first with the maximum IF of 8.955. There only were three journals with an IF >6.00, and 8 journals were with an IF < 5.00. Approximately 38.5% of the NICS papers published in the journals with IF scores over 3.00. These results indicate that it is challenging to publish papers related to NICS in high-IF journals, and there is still a lack of high-quality publications in the NICS field. Moreover, the NICS-related publications in the journals showed a dispersion, and most journals belonged to the field of neuroscience or neurological disorders. We suggest that the level and quality of research in NICS area may need some improvements and breakthrough. According to the production output and IF scores, the journal of Cerebellum, Brain stimulation and Clinical neurophysiology can be regarded as the most impactful and representative journals in this given field. It is worthwhile for researchers to continue to pay more attention to them, and some frontier papers may be published in these journals. These results means that those journals are the representative and professional journals in this given field.

### Active cooperation is necessary in NICS research

In the top 10 countries, there is only one developing country (China). Almost all of the remaining countries are developed countries in Europe and North America, accounting for 90%. As a result, European and North American countries still play the dominant roles in NICS research area. Notably, there are over 50% of top 10 institutions and high-level research authors from Italy. These results demonstrate that Italy displays the leading position in this specific field, and the researchers or institutions from Italy are the core research forces in this area.

By analysing the publications and citations, Johns Hopkins University, University College London, and University of Rome Tor Vergata are the most impactful and prolific institutions. To some extent, it indicates that these institutions are the main research forces and have a relatively high quality of published papers in this field. Celnik Pablo A from USA (citation count = 1,884), John C. Rothwell from UK (citation count = 1,741), Koch, Giacomo from Italy (citation count = 1,644) and Priori, Alberto (citation count = 1,067) are the most active and influential author. Celnik Pablo A mainly applied various NICS techniques to understand the mechanisms underlying motor learning and motor recovery after brain lesions, and on developing new strategies to enhance motor recovery after stroke (Celnik, 2015; Spampinato et al., 2020; Mooney et al., 2021, 2022). John C. Rothwell mainly explored the effects of different NICS techniques on the activation of cerebral excitability and the neural networks (Huang et al., 2005; Reis et al., 2008; Quartarone et al., 2020). Moreover, the studies of Giacomo Koch were mainly associated with the use of cerebellar TMS in stroke, Parkinson's, and other neurological diseases (Koch et al., 2007, 2019; Picazio and Koch, 2015). The research groups of Alberto Priori preferred to investigate the treatment effects of cerebellar tDCS (Ferrucci et al., 2015, 2019; Lefaucheur et al., 2017). Notably, the stable and extensive cooperations between top authors were established in the co-occurrence network maps, especially among those high-yield authors (Giacomo Koch, John C. Rothwell, and Alberto Priori). Additionally, we observed that active collaboration was established between those prolific authors or developed countries, but there was a lack of cooperation between the authors or institutions from developing countries. Thus, these high-ranking and impactful institutions and researchers can be followed, and it is necessary for authors or institutions from developing countries to strengthen cooperation with them, to promote the development of this field, and to conduct high-quality studies.

### Hot issues in NICS research

Information on keywords can help researchers identify the hot issues and emerging trends of NICS. Based on the co-occurrence and cluster analysis of keywords, the current research hotspots mainly focus on getting insights into the functional connectivity and cortical excitability between different brain regions via applying the cerebellar TMS or tDCS. It is possible to develop the NICS techniques as the new therapeutic strategies for neurological diseases. Furthermore, the keyword clusters reveal the whole knowledge structure in NICS area, and it is helpful for researchers to get an overview into this field quickly. These main topics are listed as following: Cluster #0 shown the positive effects of cerebellar tDCS on motor function and motor learning in stroke. Clusters #1 reported that the use of cerebellar CTBS could help patients with stroke to improve their speech function, cognitive impairments, eye movement and balance function. Clusters #2 and #7 mainly described the non-invasive neuromodulation and neuro electrophysiological research regarding to the cerebellar ataxia diseases. Under the Clusters #3 and #4, we can get the information about the cerebellar brain inhibition via applying the TMS in healthy people.

Burst keywords facilitate researchers to detect the emerging areas of research in a certain field quickly, and it provides directions for future research. In the past five years (2016–2021), the most important hot spots are “plasticity,” “paired associative stimulation,” “motor control,” “working memory,” “electric field,” “functional connectivity,” “scale,” “double-blind” and “transcranial direct current stimulation.” In recent years, NICS techniques have been administered in several different ways in research, such as TMS, theta burst stimulation, paired associative stimulation and tDCS (Minks et al., 2010; Ferrucci et al., 2016; Grimaldi et al., 2016). In addition, the keyword “double blind” had a strong burst, implying that an increased number of high-quality randomised controlled trials were conducted in this area. In addition, NICS research was not restricted to basic research involved in brain functional connectivity, but the clinical research is gradually increasing. To be specific, research related to NICS techniques has been increasingly performed in neurological conditions, such as ataxia, Parkinson's, and stroke (Groiss and Ugawa, 2012; Batsikadze et al., 2019; Xia et al., 2022).

Another interesting result is that “transcranial direct current stimulation” also had a strong burst in recent five years (2016–2021), instead of TMS. For instance, the tDCS studies by Doppelmayr et al. (2016) and Erfmann (2018) both demonstrated the positive effects of cerebellar tDCS on improving motor skill learning and adaptation in healthy adults. In the same year of 2016, Naro et al. (2016b) also started to devote themselves on investigating cerebellar transcranial alternating current stimulation and stroke neuroplasticity. At the begin of 2018, some researchers had shown great interests in utilising cerebellar tDCS on the improvements of motor function in stroke (Fleming et al., 2018; Kang et al., 2018). Another example of such a development was shown in studies by Solanki et al. (2021) and Rezaee et al. (2020), who investigated if cerebellar tDCS benefits the standing balance and gait performance in subjects with stroke. The developments in cerebellar tDCS research are probably due to some practical advantages of tDCS, combining the characteristics of affordable, safety and real-time (Priori et al., 2009). Furthermore, tDCS can be easily combined with other methods into research, such as electroencephalogram, functional magnetic resonance imaging (fNIRS) and functional near-infrared spectroscopy (McKendrick et al., 2015; Ulam et al., 2015; Esmaeilpour et al., 2020). For example, Rezaee et al. (2021) investigated the feasibility of applying fNIRS and EEG to guide the cerebellar tDCS treatment for chronic stroke patients. Even though Rezaee and Dutta (2019) proposed one feedforward prediction model based on computation of cerebellar lobule-specific electric field distribution to guide the selection of optimal stimulation target and dosage for cerebellar tDCS, therapeutic applications of tDCS are still in preliminary stages. Further studies are necessary to define the optimal treatment timing and dosage of cerebellar tDCS in different neurological diseases. Moreover, further studies can combine various neuroimaging or brain monitoring tools with cerebellar tDCS to reveal the underlying neuromodulation effects in neurological conditions. Thus, we predicted that the cerebellar tDCS will become the frontier trend and hot spot, and it could be a good choice for research teams in this field.

There are some limitations in our study. Firstly, we only retrieved data in the WOS database and all non-English papers were excluded. Although WOS is considered as one of the most authoritative databases, the data from in other databases such as PubMed, Scopus, and Google scholar have not been retrieved. Therefore, our results may not be comprehensive enough, and the 701 included papers only represent information from the WOS database, not all of the information in the NICS field. Secondly, only articles and reviews were included in our study, and this may not cover the full range of research undertaken in this area, such as publications in non-indexed journals, dissertations, books, or government reports.

## Conclusion

This bibliometric analysis review provides an overview on the current status and the global trend of NICS, showing that research on NICS is a promising field. The journal of Cerebellum, Brain stimulation, Clinical neurophysiology, and Neuroimage were the most influential journals in this field. Celnik, Pablo A, Rothwell, J. C and Koch, Giacomo were the most productive and influential authors. Johns Hopkins University published the NICS-related papers with the highest citation. European and North American countries prevail in the NICS research area, and they contributed the most in the number of publications and top high-cited articles in this field. Some Asian countries could be expected to make an important contribution to this field in the future, such as Japan and China. Although the broad research co-operations have been extensively among the developed countries, more active cooperation between authors, institutions and developing countries may be needed. Moreover, the current research hotspot mainly focuses on the effects of cerebellar TMS and tDCS in several neurologic conditions, such as ataxia, stroke, and Parkinson's. We expected that NICS research in the neurologic disorders will continue to grow.

## Data availability statement

The original contributions presented in the study are included in the article/supplementary material, further inquiries can be directed to the corresponding author.

## Author contributions

LH: concept, idea, research design, and writing original draught. Q-FG: data collection and data analysis. YH, H-XT, YC, C-HW, and T-YZ: contributed to revising and approving the manuscript. All authors contributed to the article and research.

## Acknowledgements

We would like to thank YH for assistance with the final version of manuscript in this study and the researchers involved who work at the Department of Rehabilitation Medicine of West China Hospital at Sichuan University.
